# Exposure to Elemental Carbon, Organic Carbon, Nitrate, and Sulfate Fractions of Fine Particulate Matter and Risk of Preterm Birth in New Jersey, Ohio, and Pennsylvania (2000–2005)

**DOI:** 10.1289/ehp.1408953

**Published:** 2015-04-24

**Authors:** Kristen M. Rappazzo, Julie L. Daniels, Lynne C. Messer, Charles Poole, Danelle T. Lobdell

**Affiliations:** 1 Department of Epidemiology, UNC Gillings School of Global Public Health, Chapel Hill, North Carolina, USA; 2 School of Community Health–College of Urban and Public Affairs, Portland State University, Portland, Oregon, USA; 3 National Health and Environmental Effects Research Laboratory, Office of Research and Development, U.S. Environmental Protection Agency, Chapel Hill, North Carolina, USA

## Abstract

**Background:**

Particulate matter ≤ 2.5 μm in aerodynamic diameter (PM_2.5_) has been consistently associated with preterm birth (PTB) to varying degrees, but roles of PM_2.5_ species have been less studied.

**Objective:**

We estimated risk differences (RD) of PTB (reported per 10^6^ pregnancies) associated with change in ambient concentrations of elemental carbon (EC), organic carbon (OC), nitrates (NO_3_), and sulfates (SO_4_).

**Methods:**

From live birth certificates from three states, we constructed a cohort of singleton pregnancies at or beyond 20 weeks of gestation from 2000 through 2005 (*n* = 1,771,225; 8% PTB). We estimated mean species exposures for each week of gestation from monitor-corrected Community Multi-Scale Air Quality modeling data. RDs and 95% confidence intervals (CIs) for four PTB categories were estimated for each exposure using linear regression, adjusted for maternal race/ethnicity, marital status, education, age, smoking, maximum temperature, ozone, and season of conception. We also adjusted for other species in multi-species models.

**Results:**

RDs varied by exposure window and outcome period. EC was positively associated with PTB after 27 and before 35 weeks of gestation. For example, for a 0.25-μg/m^3^ increase in EC exposure during gestational week 9, RD = 96 (95% CI: –20, 213) and RD = 145 (95% CI: –50, 341) for PTB during weeks 28–31 and 32–34, respectively. Associations with OCs were null or negative. RDs for NO_3_ were elevated with exposure in early weeks of gestation, and null in later weeks. RDs for SO_4_ exposure were positively associated with PTB, though magnitude varied across gestational weeks. We observed effect measure modification for associations between EC and PTB by race/ethnicity and smoking status.

**Conclusion:**

EC and SO_4_ may contribute to associations between PM_2.5_ and PTB. Associations varied according to the timing of exposure and the timing of PTB.

**Citation:**

Rappazzo KM, Daniels JL, Messer LC, Poole C, Lobdell DT. 2015. Exposure to elemental carbon, organic carbon, nitrate, and sulfate fractions of fine particulate matter and risk of preterm birth in New Jersey, Ohio, and Pennsylvania (2000–2005). Environ Health Perspect 123:1059–1065; http://dx.doi.org/10.1289/ehp.1408953

## Introduction

Particulate matter ≤ 2.5 μm in aerodynamic diameter (PM_2.5_), regulated under the Clean Air Act Amendments of 1990 (1990) as a criteria air pollutant, is a complex mixture of extremely small particles and liquid droplets. Chemical composition of PM_2.5_ varies spatially and temporally (Bell et al. 2007), as do strength of associations between PM and various health effects (Franklin et al. 2007); these variations in health effects and compositions may reflect differing toxicity of PM species. PM_2.5_ exposure has been studied with many health outcomes, among them preterm birth (PTB). PTB is a marker for fetal underdevelopment and a risk factor for further poor health outcomes (Behrman and Butler 2007; Saigal and Doyle 2008).

Although most studies of PM_2.5_ and PTB use PM_2.5_ mass as the exposure metric because of limited speciated data, four studies have examined associations between PM_2.5_ species and PTB (Brauer et al. 2008; Darrow et al. 2009; Gehring et al. 2011; Wilhelm et al. 2011). These studies have found elevations in risk or odds of PTB associated with exposure to PM_2.5_ species: elemental carbon (EC), nitrate (NO_3_), sulfate (SO_4_), and organic carbon (OC). However, null or inverse risks/odds have also been observed for these chemicals depending on study type or window of exposure examined. Two studies that examined a close corollary of EC (black carbon/soot) lacked continuous EC monitoring data for use in land-use regression models (LUR) and therefore used PM monitoring data to adjust for temporal fluctuations (Brauer et al. 2008; Gehring et al. 2011). Darrow et al. (2009) examined a number of different species but used a single monitoring location. Because all studies depended on monitoring data, study samples were limited to areas with monitors. Despite sparse data on relationships between PM_2.5_ species and PTB, there is some evidence that certain species, for example, sulfates or nitrates, may be more influential than others (Darrow et al. 2009; Wilhelm et al. 2011).

## Objectives

This study builds on previous research to examine chemical components of PM_2.5_ in relation to PTB by investigating a large study area with a wide range of PM_2.5_ levels and investigating multiple species and exposure periods. We examined associations between ambient EC, OC, SO_4_, and NO_3_ and the risk of PTB using a cohort of pregnancies reaching 20 weeks of gestation from singleton live births across 6 years and three states [Pennsylvania (PA), Ohio (OH), and New Jersey (NJ)] selected for exposure variability because they contain areas of high and low PM_2.5_ concentration. We employ the U.S. Environmental Protection Agency’s (EPA) Community Multiscale Air Quality (CMAQ) model (Hogrefe et al. 2009), which offers complete spatial coverage and daily estimated air pollutant concentrations. We estimated risk differences (RDs) and 95% confidence intervals (CIs) for 1-μg/m^3^ increases in average weekly OC, NO_3_, and SO_4_ exposures, and 0.25-μg/m^3^ increases for EC at each week of gestation and birth at four categories of preterm gestation.

## Methods

Methods are described in detail elsewhere (Rappazzo et al. 2014). Briefly, the study population was generated from live birth records provided by State Health Departments of PA, NJ, and OH. The population was restricted to singleton births with geocodable addresses and gestational age data and no recorded birth defects. To ensure that each pregnancy was entirely observable within the study period and avoid fixed-cohort bias (Strand et al. 2011), eligible pregnancies had to have achieved gestational week 20 no earlier than 1 January 2000 and gestational week 45 no later than 31 December 2005. From all birth records (*n* = 2,495,350), these restrictions led to a population of 1,940,213 pregnancies. For analysis, the population was further restricted to those with complete covariate information (*n* = 1,771,255).

Gestational age was determined by clinical estimate of gestation (CEG) as reported on birth certificates. To better elucidate severity of preterm birth and determine whether associations between preterm birth and PM_2.5_ species vary according to gestational age, PTBs were divided into four categories based on World Health Organization definitions (WHO 2013): extremely PTB (ExPTB) gestational age 20–27 weeks; very PTB (VPTB) gestational age 28–31 weeks; moderate PTB (MPTB) gestational age 32–34 weeks; and late PTB (LPTB) gestational age 35–36 weeks. Term births were between 37 and 45 completed gestational weeks.

Daily estimated concentrations of PM_2.5_ species (EC, OC, NO_3_, SO_4_) were provided by the U.S. EPA’s Atmospheric Exposure Integration Branch for 1999–2005 in 12-km grids. These estimates were constructed by Hogrefe et al. (2009) using output from CMAQ, bias-corrected with monitoring network data. Briefly, meteorological conditions and criteria pollutant emissions are input into CMAQ, which simulates atmospheric processes and estimates gridded concentrations of ambient air pollutants; grids were matched to monitoring sites and a filter applied to created baseline concentrations of PM_2.5_ species (Hogrefe et al. 2009). Adjustment factors were created (ratio of observed to modeled concentrations), spatially interpolated across the grids, and multiplied by CMAQ output to produce bias-corrected concentrations (Hogrefe et al. 2009). Maternal addresses at birth were geocoded using the ZP4 address locator program (Semaphore Corporation, Monterey, CA) and the ArcGIS online geocoding service in ArcMap 10 (ESRI, Redlands, CA). We matched addresses to CMAQ grid and concentration estimates for each day of pregnancy. Date of last menstrual period was used as “start of pregnancy” and estimated using CEG and date of birth. Exposures were averaged over fixed 7-day periods to estimate weekly average concentrations (i.e., average of days 1–7 equals concentration for week 1, average of days 8–14 equals concentration for week 2, and so on) for all pregnancies.

We identified potential confounders *a priori* based on previous literature and knowledge of factors influencing PTB and air pollution (Behrman and Butler 2007; Brauer et al. 2008; Darrow et al. 2009; Gehring et al. 2011; Knüppel and Stang 2010; Wilhelm et al. 2011). From birth certificate, we included maternal race/ethnicity (non-Hispanic white, non-Hispanic black, Hispanic, other), education (< 8th grade, some high school, high school diploma, some college, bachelor’s degree, graduate school), marital status, age at delivery (3-knot restricted quadratic spline), smoking status, and season of conception (summer: June–August; fall: September–November; winter: December–February; spring: March–May); from CMAQ we included maximum temperature (continuous, averaged daily values) and ozone concentration (continuous, averaged daily values), which has been associated with PTB and co-occurs with PM_2.5_ (Lee et al. 2013). Potential effect measure modifiers/modification (EMM) identified *a priori* included race/ethnicity (black non-Hispanic, nonblack), smoking status (smoker, nonsmoker), infant sex (male, female), and maternal parity (primiparous, multiparous). When EMM was observed (interaction terms with significance at *p* < 0.05), both marginal and stratified effect estimates are presented, because marginal effect estimates effectively standardized to population distributions reflect overall associations with PM_2.5_ species, whereas stratified effect estimates highlight potentially vulnerable subgroups for targeted intervention.

RDs were estimated using modified Poisson regression with an identity link. Poisson models produce equally valid estimates as binomial models; though Poisson models are less efficient than binomial models, they are less likely to result in nonconvergence (Spiegelman and Hertzmark 2005; Wacholder 1986; Zou 2004). In lieu of testing null hypotheses (RD = 0), we examined patterns of RD estimates and focused on their precision, thereby not raising multiple testing concerns. We estimated absolute effect measures because, with outcome severity, they are informative for public health impact and decision making. RDs are interpreted as change in PTB per 1,000,000 pregnancies for every 1-unit increase in species concentration. Number needed to treat (NNT = 1/RD) may also be calculated from RDs (Laupacis et al. 1988); NNT has a simple interpretation and improves risk communication. We used an at-risk approach in our models; all births that could have experienced birth at the weeks of interest were included in the model. For example, very preterm births (births at 28–31 weeks) were included in models of associations with extremely preterm births (births at 20–17 weeks), but extremely preterm births were not included in models of very preterm, because they were not at risk of birth at 28–31 weeks. PM_2.5_ species were modeled as continuous variables, linearity of outcome–exposure associations was examined using various coding methods (e.g., categories based on percentiles) and found to be generally linear; models using categories of species exposure were monotonic increasing or decreasing (data not shown). Exposure contrasts were set at 1 μg/m^3^ for species with an interquartile range of > 1 μg/m^3^ (OC, NO_3_, SO_4_) or at 0.25 μg/m^3^ (EC). Each species was evaluated in a single-species model and a multi-species model with all PM_2.5_ species. For comparability with previous work, we examined exposures averaged by trimester and entire pregnancy period. All statistical analyses were performed using SAS version 9.3 (SAS Institute Inc., Cary, NC).

This research was approved by the University of North Carolina at Chapel Hill’s Office of Human Research Ethics, the Pennsylvania Department of Health Bureau of Health Statistics & Research, the New Jersey Department of Health and Senior Services Institutional Review Board, and the Ohio Department of Health Human Subjects Institutional Review Board.

## Results

A total of 1,771,255 pregnancies (of potential 1,940,212) were included in adjusted analysis because they had complete covariate information. Of these, 140,987 (8%) were PTBs. Women with PTB, compared with term births, had lower educational attainment, were more often unmarried, and were more likely to be non-Hispanic black (Table 1). The study population was primarily urban, with 80% in the highest rural–urban continuum code (U.S. Department of Agriculture, Economic Research Service 2013). Women in the risk set excluded due to nongeocodable addresses were more likely to be younger, white, unmarried; have lower educational attainment; and have a PTB (~ 9% vs. 8% in analytic population) than included women. Of the women excluded due to missing covariate information, 7% were missing maternal age, whereas other covariates had low proportions of missing values (~ ≤ 1%). Proportions of missing observations were similar across PTB categories for most covariates, though maternal education and smoking status had higher proportions of missing numbers in the ExPTB group compared with other categories (e.g., smoking status missing 3% in ExPTB group vs. 1% in others).

**Table 1:** Maternal and fetal characteristics across term and preterm categories for included pregnancies in women living in OH, PA, or NJ, 2000–2005 [*n* (%)].

Characteristic	ExPTB	VPTB	MPTB	LPTB	Term births
Observations (*n*)	8,532	11,885	31,168	89,402	1,630,268
Maternal education
Graduate school	542 (6)	927 (8)	2,855 (9)	9,194 (10)	202,082 (12)
Bachelors degree	1,012 (12)	1,634 (14)	4,666 (15)	14,906 (17)	324,400 (20)
College	1,873 (22)	2,584 (22)	6,934 (22)	20,283 (23)	370,746 (23)
High school	3,172 (37)	4,182 (35)	10,670 (34)	29,357 (33)	488,712 (30)
Some high school	1,632 (19)	2,174 (18)	4,980 (16)	12,768 (14)	183,974 (11)
< 8th grade	301 (4)	384 (3)	1,063 (3)	2,894 (3)	60,354 (4)
Maternal race/ethnicity
Non-Hispanic white	4,070 (48)	6,509 (55)	18,738 (60)	58,540 (65)	1,147,612 (70)
Non-Hispanic black	3,211 (38)	3,611 (30)	7,635 (24)	16,815 (19)	222,892 (14)
Hispanic	997 (12)	1,321 (11)	3,417 (11)	9,935 (11)	176,621 (11)
Other	254 (3)	444 (4)	1,378 (4)	4,112 (5)	83,143 (5)
Maternal age at delivery (years)
< 15	124 (1)	138 (1)	282 (1)	629 (1)	7,935 (< 1)
15–19	1,207 (14)	1,397 (12)	3,213 (10)	8,412 (9)	130,052 (8)
20–24	2,088 (24)	2,765 (23)	7,107 (23)	20,052 (22)	350,059 (21)
25–29	1,997 (23)	2,746 (23)	7,513 (24)	22,912 (26)	436,453 (27)
30–34	1,814 (21)	2,785 (23)	7,522 (24)	22,447 (25)	444,216 (27)
35–39	1,030 (12)	1,615 (14)	4,362 (14)	11,988 (13)	217,260 (13)
40–44	254 (3)	415 (3)	1,100 (4)	2,790 (3)	42,480 (3)
≥ 45	18 (< 1)	24 (< 1)	69 (< 1)	172 (< 1)	1,813 (< 1)
Marital status
Married	3,844 (45)	6,010 (51)	17,395 (56)	55,307 (62)	1,113,352 (68)
Single	4,688 (55)	5,875 (49)	13,773 (44)	34,095 (38)	516,916 (32)
Maternal smoking status
No	6,639 (78)	9,219 (78)	24,446 (78)	72,374 (81)	1,385,050 (85)
Yes	1,893 (22)	2,669 (22)	6,722 (22)	17,028 (19)	245,218 (15)
Infant sex
Male	4,460 (52)	6,314 (53)	16,638 (53)	47,666 (53)	830,821 (51)
Female	4,068 (48)	5,571 (47)	14,529 (47)	41,735 (47)	799,432 (49)
Missing	4 (0)	0 (0)	1 (0)	1 (0)	15 (0)
Season of conception
Summer	1,952 (23)	2,816 (24)	7,224 (23)	20,904 (23)	385,555 (24)
Fall	2,324 (27)	3,269 (28)	8,688 (28)	24,703 (28)	457,486 (28)
Winter	2,316 (27)	3,193 (27)	8,360 (27)	23,999 (27)	436,947 (27)
Spring	1,940 (23)	2,607 (22)	6,896 (22)	19,796 (22)	350,280 (21)
Parity
Primiparous	4,291 (50)	5,671 (48)	14,168 (45)	37,373 (42)	651,940 (40)
Multiparous	4,196 (49)	6,156 (52)	16,875 (54)	51,768 (58)	974,242 (60)
Missing	45 (1)	58 (0)	125 (< 1)	261 (0)	4,086 (0)

Average weekly pollutant concentrations were similar across categories of PTB (see Supplemental Material, Table S1). Temporal correlation coefficients ranged from 0.76 to 0.50 for EC, 0.75 to 0 for OC, 0.70 to –0.45 for NO_3_, and 0.64 to –0.45 for SO_4_. Correlations between species were high for EC–OC (~ 0.80) and OC–NO_3_ (~ 0.59) and moderate for EC–NO_3_ (~ 0.38), OC–SO_4_ (~ –0.21), and NO_3_–SO_4_ (~ –0.43). EC and SO_4_ were not correlated (~ –0.07).

**Figure 1:**
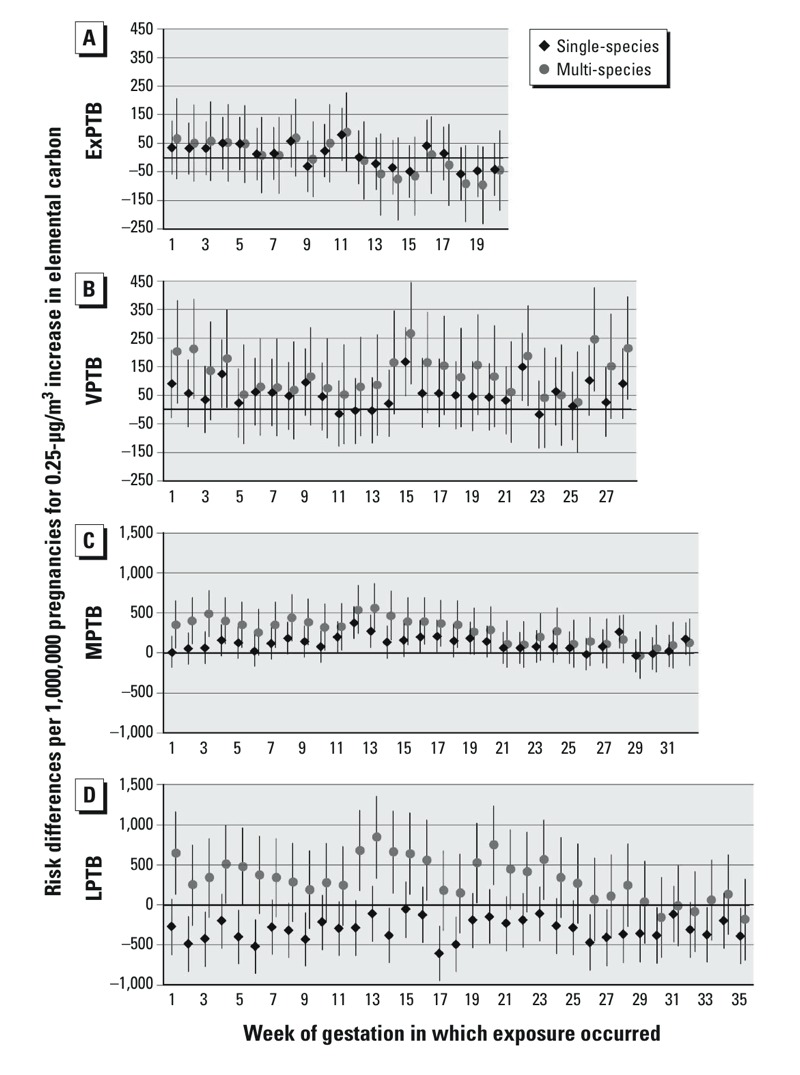
Risk differences per 1,000,000 pregnancies for 0.25-μg/m^3^ increases in EC. Single-species models were adjusted for maternal education, race/ethnicity, marital status, age at delivery, smoking, season of conception, maximum temperature, and O_3_. Multi-species models are also adjusted for OC, NO_3_, and SO_4_. (*A*) ExPTB: birth at 20–27 weeks, (*B*) VPTB: birth at 28–31 weeks, (*C*) MPTB: birth at 32–34 weeks, and (*D*) LPTB: birth at 35–36 weeks of gestation.

PTB RDs associated with a 0.25-μg/m^3^ increase in EC, with exposure at each week of gestation, are shown in Figure 1. For EC exposure, ExPTB RDs were null for exposure at all gestational weeks, with similar estimates from single- and multi-species models. For VPTB, single-species model RDs were slightly elevated at some points in mid-pregnancy, though many weeks were near null. However, EC RDs were generally higher in multi-species models. For MPTB, single-species RDs were elevated with exposure to EC at gestational weeks 4 and 5, and consistently positive from week 7 through week 21. Multi-species model RDs were positive for exposures in weeks 1–21, and higher than corresponding RDs from the single-species models. For LPTB, RDs from single-species models were consistently negative or null, whereas EC RDs from multi-species models were positive for exposures during weeks 1–24.

**Figure 2:**
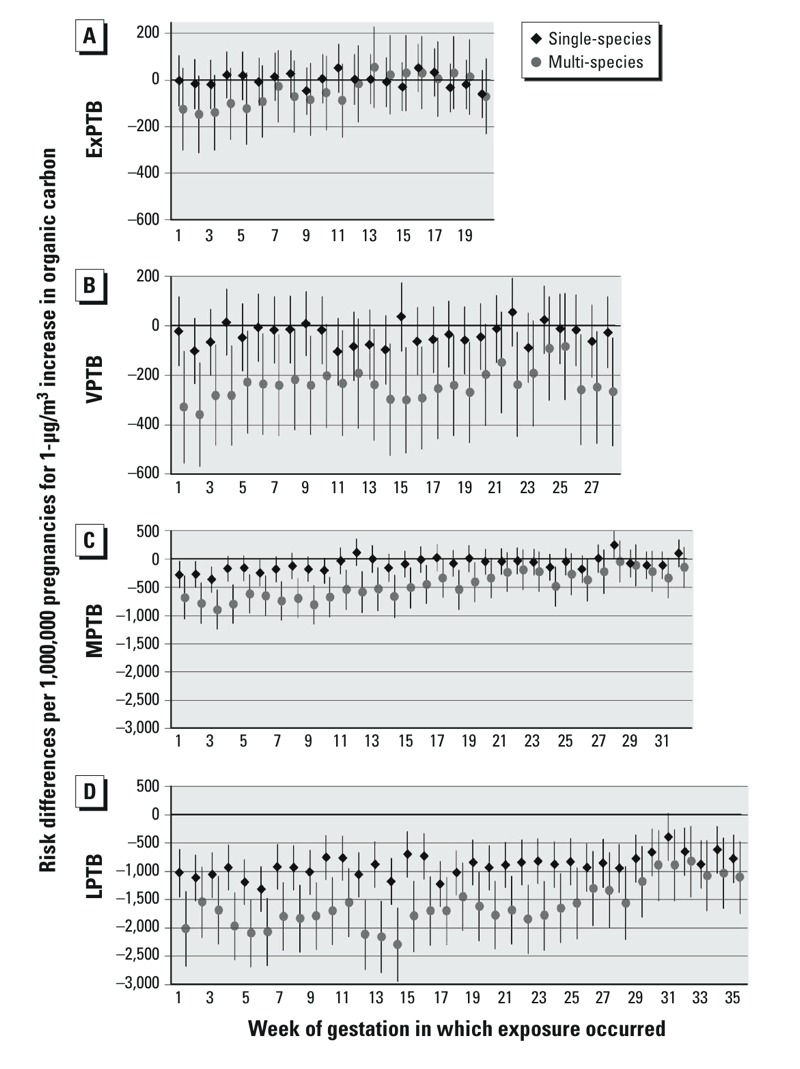
Risk differences per 1,000,000 pregnancies for 1-μg/m3 increases in OC. Single-species models were adjusted for maternal education, race/ethnicity, marital status, age at delivery, smoking, season of conception, maximum temperature, and O3. Multi-species models are also adjusted for OC, NO3, and SO4. (A) ExPTB: birth at 20–27 weeks, (B) VPTB: birth at 28–31 weeks, (C) MPTB: birth at 32–34 weeks, and (D) LPTB: birth at 35–36 weeks of gestation.

PTB RDs for a 1-μg/m^3^ increase in OC with exposures at each week of gestation are shown in Figure 2. In single and multi-species models associations of OC were generally null for ExPTB. For VPTB, OC RDs were generally null in single-species models, but were negative in multi-species models. For MPTB, single-species RDs were null, whereas multi-species RDs were negative for exposures in weeks 1–27. For LPTB, single-species RDs were negative for exposures across all weeks of gestation. In multi-species models, OC RDs were more negative than in single-species models for exposures at weeks 1–25, after which RDs were similar to those observed in single-species models.

**Figure 3:**
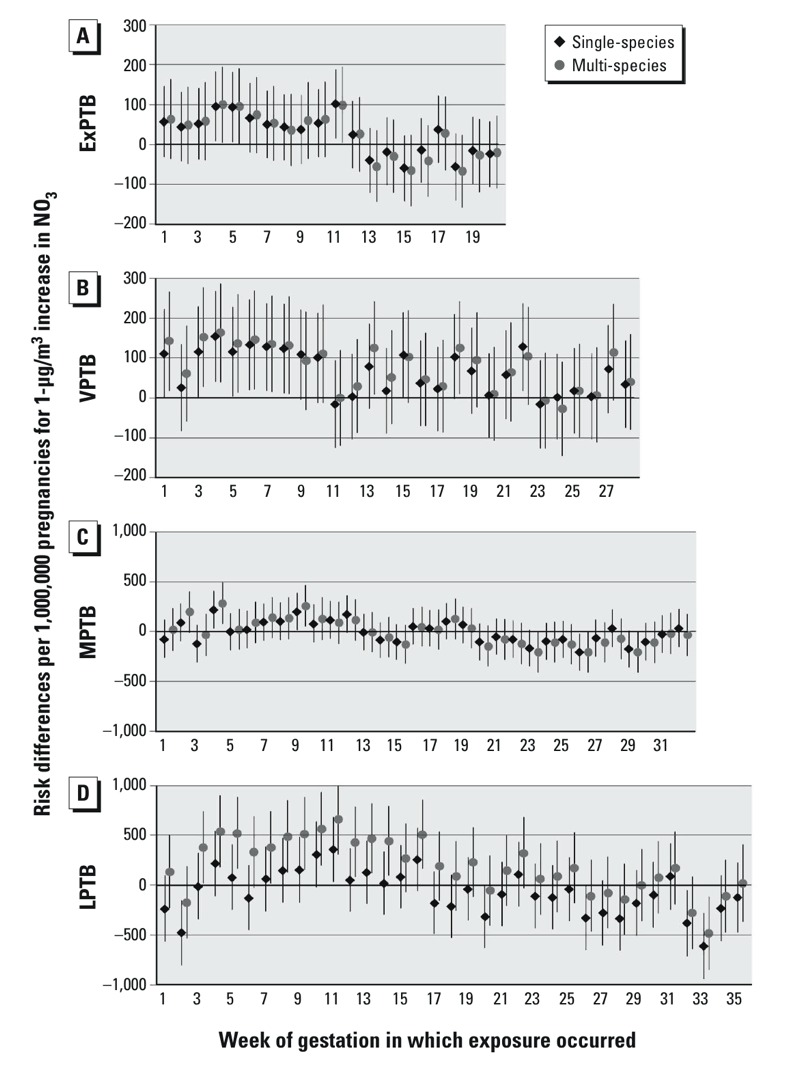
Risk differences per 1,000,000 pregnancies for 1-μg/m^3^ increases in NO_3_. Single-species models were adjusted for maternal education, race/ethnicity, marital status, age at delivery, smoking, season of conception, maximum temperature, and O_3_. Multi-species models are also adjusted for OC, NO_3_, and SO_4_. (*A*) ExPTB: birth at 20–27 weeks, (*B*) VPTB: birth at 28–31 weeks, (*C*) MPTB: birth at 32–34 weeks, and (*D*) LPTB: birth at 35–36 weeks of gestation.

PTB RDs for a 1-μg/m^3^ increase in NO_3_ with exposures at each week of gestation are shown in Figure 3. For NO_3_ exposure, in both single and multi-species, RDs for ExPTB were consistently elevated at gestational weeks 1–11, then near null or negative. For VPTB, single-species associations were elevated for exposure in weeks 3–10, then sporadically positive over later weeks; multi-species RDs were similar to single-species RDs. For MPTB and LPTB, single-species RDs were generally null or slightly elevated in early weeks, and null afterward with some negative risks at the latest weeks. With multi-species models, RDs for NO_3_ were similar to single-species associations for MPTB, but became more positive for LPTB, particularly in earlier weeks of exposure.

**Figure 4:**
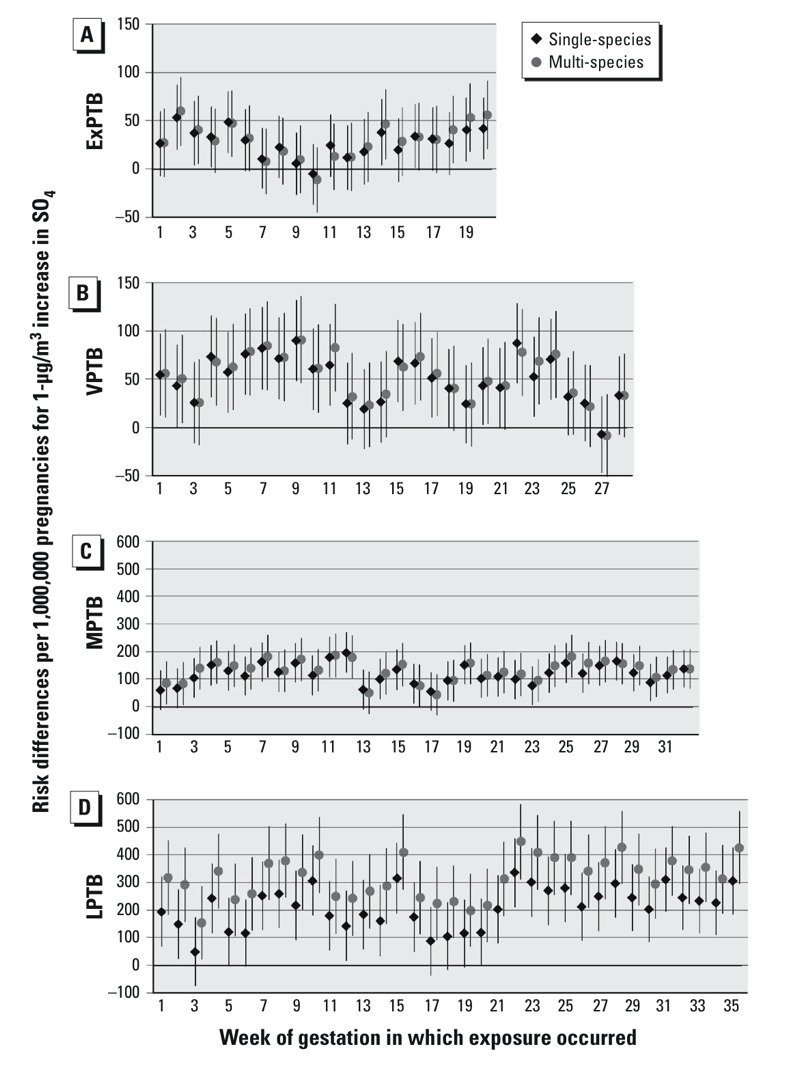
Risk differences per 1,000,000 pregnancies for 1-μg/m3 increases in SO4. Single-species models were adjusted for maternal education, race/ethnicity, marital status, age at delivery, smoking, season of conception, maximum temperature, and O3. Multi-species models are also adjusted for OC, NO3, and SO4. (A) ExPTB: birth at 20–27 weeks, (B) VPTB: birth at 28–31 weeks, (C) MPTB: birth at 32–34 weeks, and (D) LPTB: birth at 35–36 weeks of gestation.

PTB RDs for a 1-μg/m^3^ increase in SO_4_ with exposures at each week of gestation are shown in Figure 4. For SO_4_ exposure in single species models, ExPTB exhibits positive RDs before week 7 and after week 13, with null associations for weeks 8–12; multi-species model RDs were similar to single-species model RDs. RDs for VPTB, MPTB, and LPTB were generally elevated across all weeks of gestation in single- and multi-species models.

**Figure 5:**
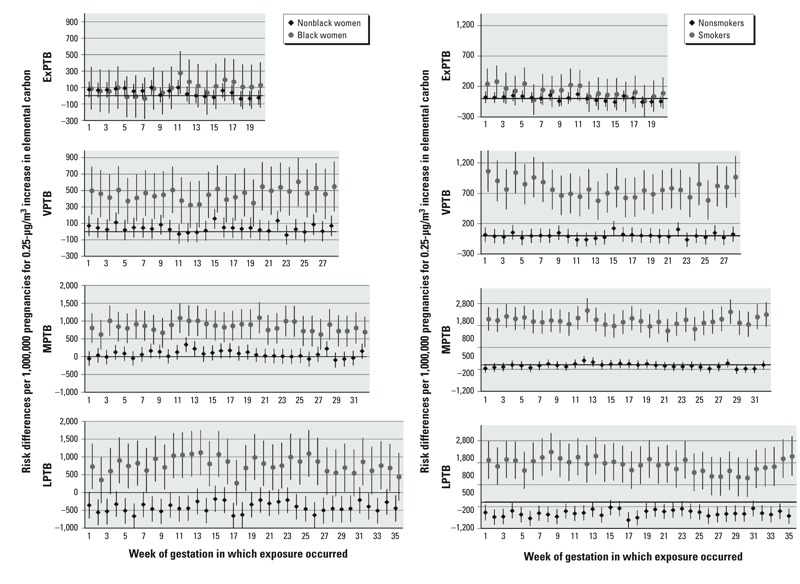
Effect measure modification by black race/ethnicity (left) and smoking status (right) for elemental carbon. Models were adjusted for maternal education level, marital status, age at delivery, smoking status (race/ethnicity models), race/ethnicity (smoking status models), season of conception, maximum temperature, and co-occurring ozone. ExPTB: birth at 20–27 weeks, VPTB: birth at 28–31 weeks, MPTB: birth at 32–34 weeks, and LPTB: birth at 35–36 weeks of gestation. Weeks where interaction terms have *p *< 0.05 are: ExPTB: none; VPTB: all; MPTB: all; LPTB: all.

No EMM (interaction term *p* < 0.05) was observed for any species by infant sex or maternal parity, data not shown. RDs for EC exposure and VPTB, MPTB, and LPTB were higher than would be expected on an additive scale for women of black race/ethnicity and women who smoked during pregnancy (Figure 5). There was evidence for modification of effect estimates for OC exposures with race/ethnicity and smoking status, following similar patterns for EC estimates (see Supplemental Material, Figure S1). However, EMM for OC was less consistent across exposure categories than EMM for EC; where interaction terms met criteria at most or all weeks of exposure for EC, there was considerable variation in OC interaction terms across exposure weeks and outcome groups. For NO_3_, potential EMM was observed only for maternal smoking status in LPTB outcomes with exposure in early gestation (see Supplemental Material, Figure S2). There was limited to no evidence for EMM for SO_4_ exposures (see Supplemental Material, Figure S3).

For trimester and pregnancy exposures, associations were generally positive for NO_3_ and SO_4_ and negative for OC across exposure windows, whereas associations with EC were generally positive for first-trimester and negative for entire-pregnancy windows (see Supplemental Material, Table S2). Third-trimester and entire-pregnancy exposures should be interpreted with caution because length of exposure period varies based on gestational age.

## Discussion

We found PTB risk associated with PM_2.5_ varies by species, with potential for different associations at early versus late PTB and during different windows of exposure. EC had the strongest associations in VPTB and MPTB, particularly with exposure windows before the 23rd week of gestation and across single- and multi-species models. Associations for EC and LPTB diverged between single- and multi-species models, with negative RDs in single-species models and positive RDs in multi-species models. EC also demonstrated EMM with race/ethnicity (black non-Hispanic vs. nonblack) and smoking status wherein RDs among black women were higher than among nonblack women, with smoking status following similar patterns. We observed negative RDs with OC, but only in LPTB or multi-species models. Because it is unlikely that OCs have a protective health effect, this may be attributable to model error (e.g., in multi-species models where EC has a higher association, OC has a lower association, possibly an artifact of high correlation). It is also possible that due to correlation, the presence of both EC and OC in models creates a sparse data problem. If so, we would expect to see associations moving drastically away from the null, leading to biased RDs and interpretations. However, these drastic shifts do not seem to be the case for EC and OC RDs in our analysis. Other potential explanations for negative RDs observed include interactions between EC and OC and nonlinear associations. NO_3_ was associated with increased RDs of PTB with exposures in weeks of the first trimester with earlier PTB categories. SO_4_ shows increased RDs for PTB across categories of gestation and with most exposure windows. SO_4_ appears to have a role in risk of PTB, though no particular windows of vulnerability emerged from our analysis.

Though mechanisms of action for PM_2.5_ species on PTB are poorly understood at present, likely contenders are inflammatory or oxidative stress processes. PM_2.5_ exposure has been linked to increased markers of systemic inflammation in humans and changes in placental morphology in mice, which may be a consequence of inflammation (Backes et al. 2013; Veras et al. 2008). Changes in the placenta may lead to inadequate placental perfusion or impaired nutrient exchange (Kannan et al. 2006). Inflammation may also lead to the creation of reactive oxygen species, which can cause cell damage, DNA damage, disruption of cellular processes, irreversible protein modifications, or alterations in cellular signaling (U.S. EPA 2009). It is not known whether these processes would lead to PTB; however, they are alterations of normal function and may disrupt normal processes of gestation.

Each species has properties that may cause harm, though again these pathways are poorly understood for pregnancy outcomes. EC may directly induce a health response or may bear other toxic chemicals that induce inflammation (Ritz and Wilhelm 2008). EMM observed with race/ethnicity and smoking status may indicate priming (an initial insult that makes a secondary insult more effective) by exposure to the pollutants in cigarette smoke and the social stressors we believe race/ethnicity represents. Associations observed with OC exposures should be interpreted with caution because the modeling of OCs is restricted to primary OC, which is directly emitted to the atmosphere from a source. Secondary OC, formed by atmospheric reactions during transport, can comprise a substantial fraction of OC mass (U.S. EPA 2009), but was not included in the CMAQ model used in our analysis due to uncertainties with estimating the fraction using chemical transport models. This gives the OC concentrations an unexpectedly high correlation with EC (which is formed only through primary processes), and associations may be unduly influenced by this correlation. The epidemiologic and toxicological literature lacks discussion of the role of NO_3_ and SO_4_ on birth outcomes. However, some possibilities may be extrapolated from other literature indicating that they mediate inflammatory processes (Bind et al. 2012). The area of PM_2.5_ species and PTB has received limited attention, though it is likely that toxicity of PM_2.5_ species influence PM_2.5_’s effects on PTB.

To our knowledge, no other studies of PM_2.5_ species have examined EMM. We believe our finding of high RDs for women of black race/ethnicity compared with nonblack women is not likely to be a biological effect of race/ethnicity, but rather a priming effect of factors that race/ethnicity represents [e.g., stress due to institutionalized racism (Jones 2000; Nuru-Jeter et al. 2009)]. In this situation such factors would deplete women’s resources for dealing with EC insults; therefore, EC would have a worse impact on health. In other words, social stressors related to race/ethnicity in the United States may create an “allostatic load” in which a woman has long-term biological dysregulation due to continuous or frequently occurring presence of stressors (McEwen and Seeman 1999). This then prevents women from responding biologically appropriately to further stressors, including air pollutants. Smoking may work in a similar, though more direct, manner. Particulates in cigarette smoke offer an initial insult, which may overwhelm a woman’s protective/coping processes, and EC exposure adds an additional insult that then has fewer barriers to adverse effects. These pathways are plausible, but there has been little research on this topic.

Presently, four studies have been published on the influence of PM_2.5_ species on PTB, with a variety of study designs, exposures, and exposure assessments between them (Brauer et al. 2008; Darrow et al. 2009; Gehring et al. 2011; Wilhelm et al. 2011). Two studies used LUR methods to estimate soot or black carbon exposures. Brauer et al. (2008) found no association of black carbon with entire-pregnancy exposures, and Gehring et al. (2011) found positive odds ratios with entire-pregnancy and last-month exposures. As Brauer et al. (2008) recount in their study, the LUR model performed poorly in evaluation tests and used PM_2.5_ data for temporal adjustment, because only annual black carbon data were available. As reported in their publication, Gehring et al. (2011) did not account for spatial variation of soot/PM_2.5_ between 1999/2000, years when the environmental data were available, and 1996/1997, years when pregnancies were studied. Our study shares a cohort study design with these studies but uses different exposure assessment methods. Although the LUR models offered advantages in detection of spatial heterogeneity of air pollutants, our use of bias-corrected CMAQ model offered other benefits. Our study was large and included a broad population and did not need to be limited to a monitor-rich area. Our CMAQ model was also constructed using data covering the entirety of pregnancies in our study population.

Two further studies each examined a variety of PM_2.5_ species. In a time-series study of the Atlanta, Georgia, area, Darrow et al. (2009) estimated positive risk ratios for NO_3_ and SO_4_ with exposure in the first month of pregnancy and for SO_4_ and EC with a 1-week lag from birth. OCs had null RDs for all exposure windows examined. Given differences in study design and exposure assessment (use of a single monitor for PM_2.5_ species ascertainment), our results are fairly congruent with the results from Darrow et al. (2009). Differences between results do occur; for example, with EC exposures in early pregnancy, we found elevated RDs, though not for all PTB categories. Wilhelm et al. (2011) used a case–control design with entire pregnancy exposures and found elevated odds ratios with EC, OC, and ammonium nitrate exposure in single-species models and ammonium nitrate in multi-species models. Odds ratios were null for ammonium sulfate in single-species models and inverse in multi-species models. Wilhelm et al.’s (2011) use of a dense monitoring network for species analyses may better characterize PM_2.5_ species spatial heterogeneity than CMAQ models. Wilhelm et al. (2011) reported on whole-pregnancy exposure because there was limited temporal variation in exposure, whereas we were able to examine weekly exposure windows throughout pregnancy. This investigation allowed us to examine associations with exposure at each specific week of gestation, which may help identify the most relevant exposure time windows for targeted mechanistic research.

Our study also diverges from previous literature with examination of EMM, RD as the measures of effect, and use of refined PTB categories. Broadly defined, PTB captures a 4-month period across pregnancy wherein development occurs rapidly. By examining more refined categories of PTB, rather than a single outcome, we investigate associations specific to each gestational age. The etiology of PTB may vary by gestational week because fetal development and vulnerabilities shift rapidly across the 4-month period comprising PTB. Our analysis supported this, as we observed different associations based on PTB category, which collapsing PTB into one category may have masked.

To better inform both etiology and policy research, we took advantage of our large population to examine potential EMM, providing insight into possible vulnerable populations. Estimating NNTs for risk communication is also a simple endeavor. For example, at week 5 of gestation, NNTs for EC and VPTB correspond to 8,936 for nonblack and 1,970 for black women; meaning for every 0.25-μg/m^3^ increase in ambient EC concentrations for 8,936 nonblack or 1,970 black pregnant women, one VPTB occurs (assuming causality). For public health regulatory processes to work best, understanding of risk for both the aggregate and vulnerable subpopulations is needed.

As with many studies of air pollution, classification of exposure is imperfect. Even with bias correction, the models used to estimate pollutant concentrations are limited. This is particularly true with PM_2.5_ species, for which monitoring remains sparse, and monitoring of particular species may be inconsistent across sites. For example, CMAQ models (uncorrected) are known to underperform for prediction of OC concentrations; bias correction can improve OC estimates, but high uncertainty for predictions remains because the distance between monitors is large and the same measurement methods are not used at all sites (Hogrefe et al. 2009). In addition, the 12-km grids used here will smooth over important intraurban variation in exposure. Secondary pollutants such as NO_3_ and SO_4_ are typically well correlated on a regional scale, whereas EC is more spatially diverse, with most impacts near combustion sources. Athough all models have performance issues to some degree, the concentration estimates produced by CMAQ models allow for the estimation of health effects across large areas/populations, whereas the use of only monitoring networks is much more constrained to regions geographically close to monitors. Beyond limitations specific to the use of models, potential exposure classification issues include use of ambient rather than personal measures of PM_2.5_ species and using a single residential point for exposure assignment rather than a profile of where a woman’s time is spent—including indoor versus outdoor exposure and the assumption that women had a single residence throughout pregnancy. Though these factors are likely nondifferential by outcome, their consequences may be complicated, leading to responses that may be biased toward or away from the null depending on whether estimated exposures were lower or higher than true exposures or whether these factors are differential by confounders such as socioeconomic status. Although having individual-level exposures would be beneficial, we can interpret the observed results as estimated effects of ambient exposures, which are most likely to be affected by changes to air pollution regulations.

This study identified associations between average weekly exposure to EC, OC, NO_3_, and SO_4_ during gestation and risk of four categories of PTB for women residing in OH, PA, or NJ from 2000 through 2005. EC and SO_4_, among the best characterized of the PM_2.5_ species, had the most consistent associations with risk of PTB in both single-and multi-species models. Differences existed not only between PM_2.5_ species, but also with different windows of exposure and PTB at specific gestational ages. These results suggest diverse periods of action for the species of PM, along with differing windows of vulnerability for various categories of PTB. Growth in our understanding of these complex relations will require future studies of particulate matter and its components to incorporate careful assessment of exposure timing and refined definitions of preterm gestational age.

## Supplemental Material

(851 KB) PDF
