# Supplementary material for: Exposure to Elemental Carbon, Organic Carbon, Nitrate, and Sulfate Fractions of Fine Particulate Matter and Risk of Preterm Birth in New Jersey, Ohio, and Pennsylvania (2000–2005)
Source: Environ Health Perspect. 2015 Apr 24;123(10):1059–65. doi: 10.1289/ehp.1408953 (PMC4590756; doi:10.1289/ehp.1408953)
Supplement: (851 KB) PDF [file ehp.1408953.s001.acco.pdf]

Environ Health Perspect
doi: 10.1289/ehp.1408953

Note to Readers: *EHP* strives to ensure that all journal content is accessible to all readers. However, some figures and Supplemental Material published in *EHP* articles may not conform to 508 standards due to the complexity of the information being presented. If you need assistance accessing journal content, please contact ehp508@niehs.nih.gov. Our staff will work with you to assess and meet your accessibility needs within 3 working days.

Supplemental Material

Exposure to Elemental Carbon, Organic Carbon, Nitrate, and Sulfate Fractions of Fine Particulate Matter and Risk of Preterm Birth in New Jersey, Ohio, and Pennsylvania (2000–2005)

Kristen M. Rappazzo, Julie L. Daniels, Lynne C. Messer, Charles Poole, and Danelle T. Lobdell

Table of Contents

Table S1. Descriptive statistics for particulate matter species (μg/m^3) exposure concentrations, averaged over all weeks of exposure.

Figure S1: Effect measure modification by race/ethnicity (left) and smoking status (right) for organic carbon. Models are adjusted for maternal education level, marital status, age at delivery, smoking status (race/ethnicity models), race/ethnicity (smoking status models), season of conception, maximum temperature and co-occurring ozone. Weeks where interaction terms have $p<0.05$ are: ExPTB: none; VPTB: smoking 1-8, 10-12, 14, 18-21, 24, 26-28, race/eth 2, 10, 20-21, 23-28; MPTB: smoking all, race/eth 1, 3, 4, 6, 10-11, 13-15, 17-24, 26, 31; LPTB: smoking 1-15, 16, race 1, 3-5, 7-16, 13-14, 27.

Figure S2: Effect measure modification by race/ethnicity (left) and smoking status (right) for NO_3. Models are adjusted for maternal education level, marital status, age at delivery, smoking status (race/ethnicity models), race/ethnicity (smoking status models), season of conception, maximum temperature and co-occurring ozone. Weeks where interaction terms have $p<0.05$ are:

ExPTB: none; VPTB: smoking 16, 22 race/eth 19; MPTB: none; LPTB: smoking 1-11, 13-14, 27, 16, race 2, 8, 10-14, 18-19.

Figure S3: Effect measure modification by race/ethnicity (left) and smoking status (right) for SO_4. Models are adjusted for maternal education level, marital status, age at delivery, smoking status (race/ethnicity models), race/ethnicity (smoking status models), season of conception, maximum temperature and co-occurring ozone. Weeks where interaction terms have $p<0.05$ are: ExPTB: smoking 2-4, 20, race/eth 4; VPTB: none; MPTB: smoking 15; LPTB: smoking 2, 4, 7, 10, 12-14 race 15-22.

Table S2: Risk differences per 1,000,000 pregnancies with trimester end entire pregnancy length exposure windows. Exposure contrasts: 0.25$\mu g/m^3$ for elemental carbon (EC), 1$\mu g/m^3$ for organic carbon (OC), 1$\mu g/m^3$ for nitrates (NO_3), and 1$\mu g/m^3$ for sulfates (SO_4).

Table S1. Descriptive statistics for particulate matter species ($\mu g/m^3$) exposure concentrations, averaged over all weeks of exposure.

Statistic	ExPTB	VPTB	MPTB	LPTB	Term
**Elemental Carbon**					
Min	0.07	0.06	0.04	0.04	0.02
25th	0.37	0.37	0.36	0.35	0.35
50th	0.51	0.51	0.49	0.48	0.47
75th	0.72	0.71	0.69	0.67	0.65
Max	4.08	4.09	4.39	4.72	5.04
Mean	0.61	0.6	0.58	0.56	0.55
SD	0.37	0.36	0.35	0.34	0.32
IQR	0.34	0.34	0.33	0.32	0.31
**Organic Carbon**					
Min	0.20	0.17	0.12	0.10	0.06
25th	1.12	1.11	1.09	1.07	1.06
50th	1.61	1.59	1.56	1.52	1.51
75th	2.33	2.31	2.26	2.20	2.18
Max	11.65	11.69	12.49	12.85	12.61
Mean	1.90	1.88	1.83	1.79	1.77
SD	1.14	1.12	1.10	1.07	1.05
IQR	1.20	1.22	1.22	1.21	1.09
**Nitrate**					
Min	<0.01	<0.01	<0.01	<0.01	<0.01
25th	0.47	0.47	0.47	0.46	0.45
50th	1.43	1.43	1.40	1.39	1.36
75th	2.75	2.73	2.68	2.65	2.63
Max	11.91	11.95	12.48	12.98	13.38
Mean	1.81	1.80	1.77	1.75	1.74
SD	1.58	1.57	1.55	1.54	1.53
IQR	2.28	2.26	2.22	2.19	2.18
**Sulfate**					
Min	0.90	0.90	0.84	0.80	0.69
25th	2.81	2.79	2.79	2.77	2.78
50th	4.48	4.46	4.51	4.51	4.54
75th	7.7	7.66	7.76	7.78	7.82
Max	32.95	33.89	35.51	37.28	41.38
Mean	5.74	5.72	5.77	5.77	5.78
SD	3.95	3.95	3.99	4.00	3.98
IQR	4.75	4.78	4.91	4.98	4.93

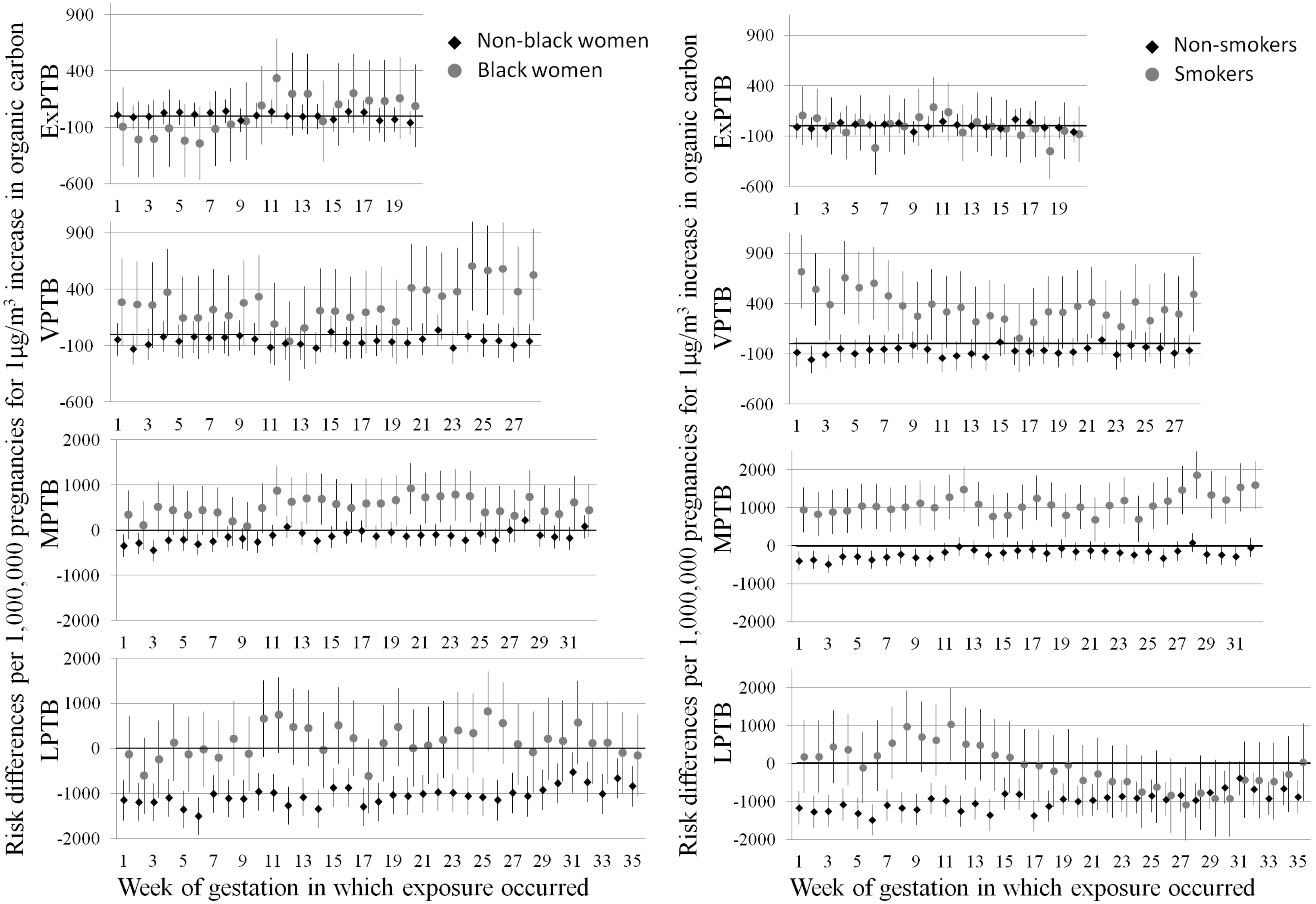

Figure S1: Effect measure modification by race/ethnicity (left) and smoking status (right) for organic carbon. Models are adjusted for maternal education level, marital status, age at delivery, smoking status (race/ethnicity models), race/ethnicity (smoking status models), season of conception, maximum temperature and co-occurring ozone. Weeks where interaction terms have $p<0.05$ are: ExPTB: none; VPTB: smoking 1-8, 10-12, 14, 18-21, 24, 26-28, race/eth 2, 10, 20-21, 23-28; MPTB: smoking all, race/eth 1, 3, 4, 6, 10-11, 13-15, 17-24, 26, 31; LPTB: smoking 1-15, 16, race 1, 3-5, 7-16, 13-14, 27.

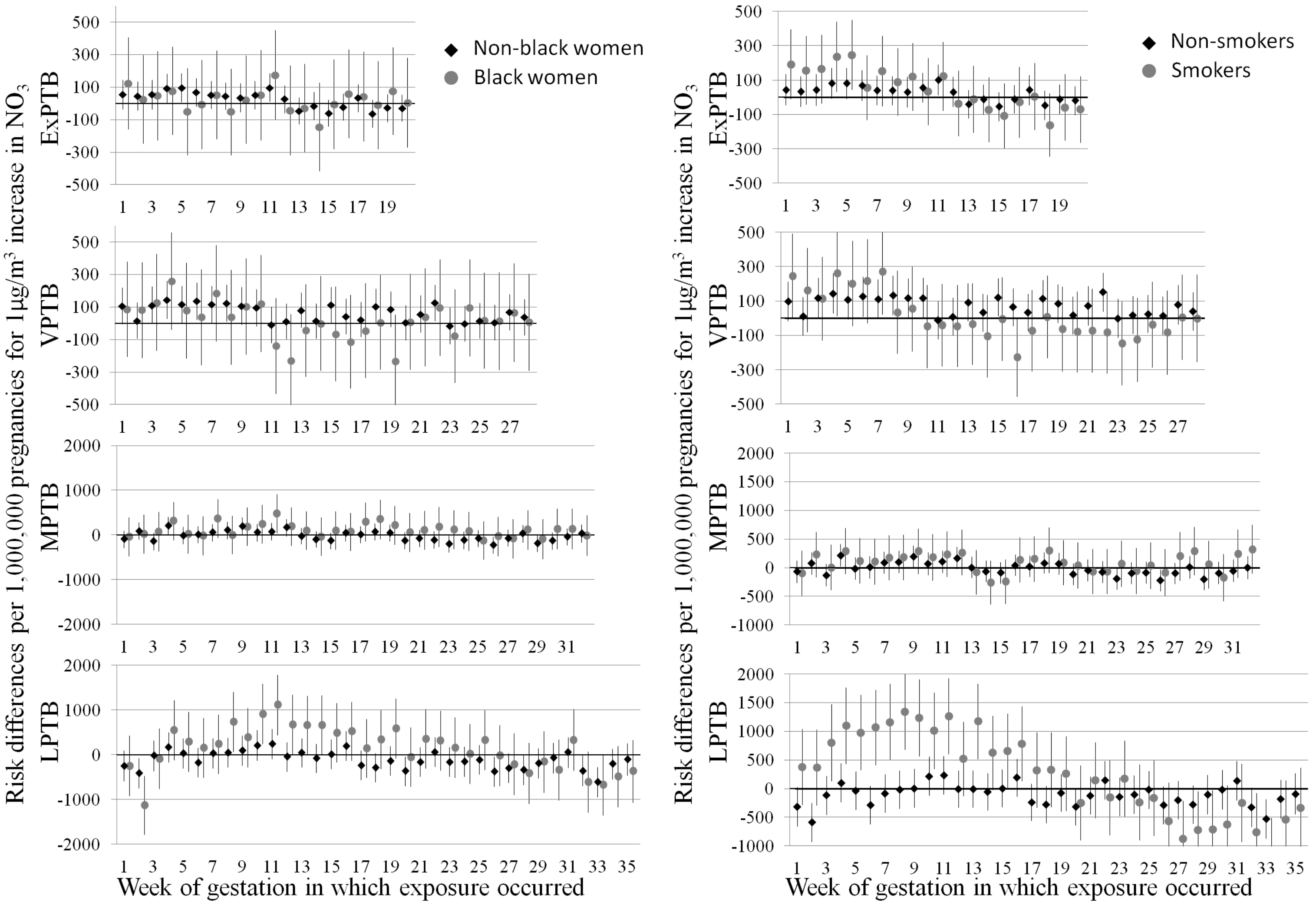

Figure S2: Effect measure modification by race/ethnicity (left) and smoking status (right) for NO_3. Models are adjusted for maternal education level, marital status, age at delivery, smoking status (race/ethnicity models), race/ethnicity (smoking status models), season of conception, maximum temperature and co-occurring ozone. Weeks where interaction terms have $p<0.05$ are: ExPTB: none; VPTB: smoking 16, 22 race/eth 19; MPTB: none; LPTB: smoking 1-11, 13-14, 27, 16, race 2, 8, 10-14, 18-19.

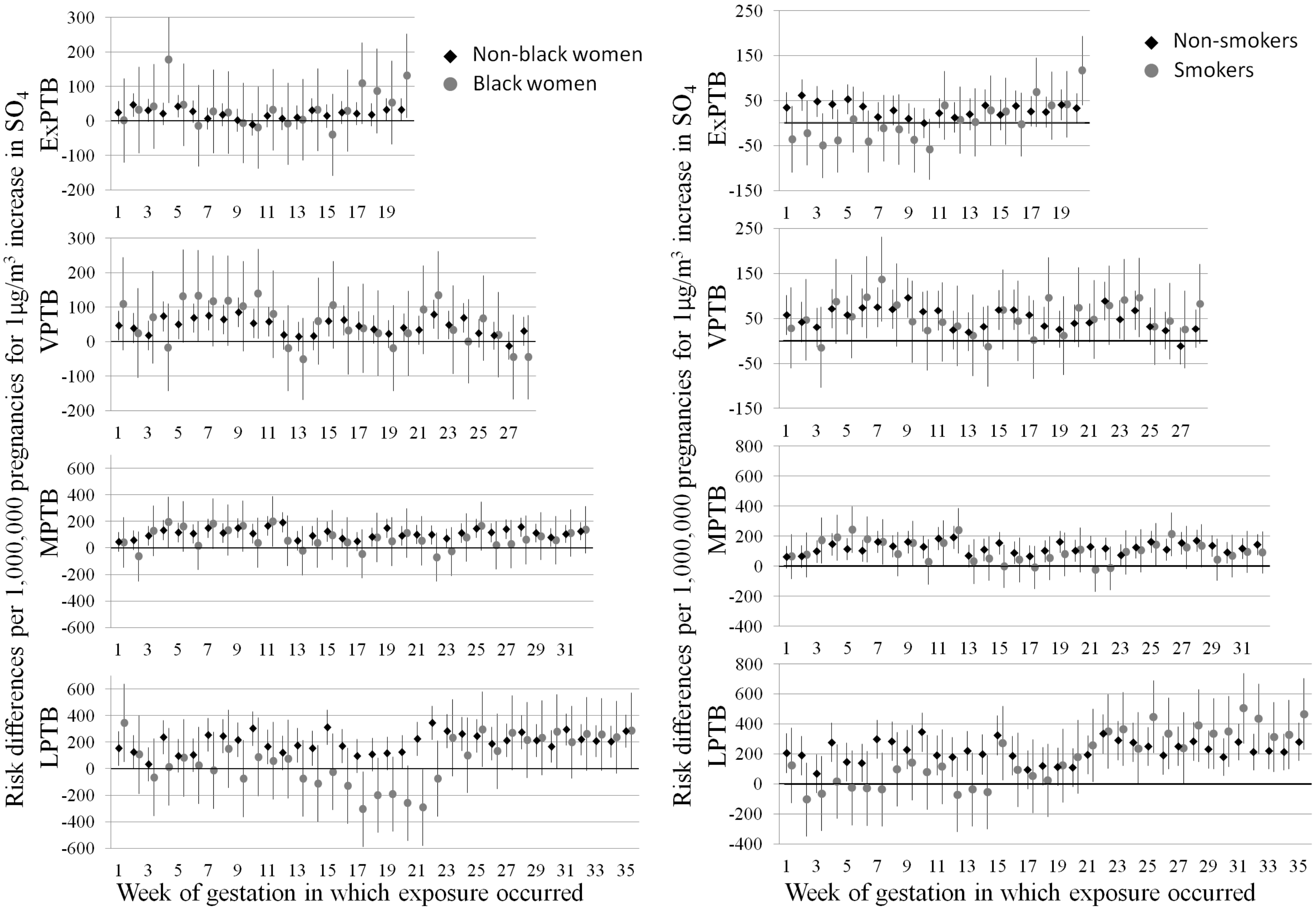

Figure S3: Effect measure modification by race/ethnicity (left) and smoking status (right) for SO_4. Models are adjusted for maternal education level, marital status, age at delivery, smoking status (race/ethnicity models), race/ethnicity (smoking status models), season of conception, maximum temperature and co-occurring ozone. Weeks where interaction terms have $p<0.05$ are: ExPTB: smoking 2-4, 20, race/eth 4; VPTB: none; MPTB: smoking 15; LPTB: smoking 2, 4, 7, 10, 12-14 race 15-22.

Table S2: Risk differences per 1,000,000 pregnancies with trimester end entire pregnancy length exposure windows. Exposure contrasts: 0.25μg/m^3 for elemental carbon (EC), 1μg/m^3 for organic carbon (OC), 1μg/m^3 for nitrates (NO_3), and 1μg/m^3 for sulfates (SO_4).

**Exposure Window**	**EC, only**	**EC, all species**	**OC, only**	**OC, all species**
ExPTB				
Trimester 1	76 (-41, 192)	96 (-68, 259)	31 (-118, 180)	-154 (-355, 48)
Trimester 2	-62 (-184, 60)	-157 (-330, 15)	-26 (-181, 130)	7 (-203, 217)
Entire Pregnancy	-230 (-441, -20)	*	31 (-166, 228)	*
VPTB				
Trimester 1	120 (-28, 268)	131 (-74, 336)	-67 (-262, 129)	-425 (-689, -162)
Trimester 2	115 (-38, 268)	132 (-78, 343)	-60 (-263, 142)	-360 (-628, -91)
Trimester 3	72 (-111, 254)	167 (-81, 415)	11 (-245, 268)	-195 (-519, 129)
Entire Pregnancy	-268 (-509, -27)	-239 (-543, 64)	-64 (-317, 188)	57 (-280, 395)
MPTB				
Trimester 1	191 (-33, 414)	290 (-20, 599)	-349 (-645, -52)	-993 (-1397, -590)
Trimester 2	88 (-140, 316)	-8 (-323, 307)	-244 (-551, 63)	-500 (-911, -89)
Trimester 3	-52 (-285, 182)	-75 (-404, 253)	-87 (-424, 250)	-225 (-678, 229)
Entire Pregnancy	-472 (-790, -153)	-486 (-897, -74)	-355 (-730, 20)	-155 (-653, 342)
LPTB				
Trimester 1	-464 (-890, -38)	334 (-252, 920)	-2007 (-2581, -1433)	-3245 (-4016, -2474)
Trimester 2	-117 (-553, 319)	266 (-334, 865)	-1727 (-2317, -1138)	-2969 (-3754, -2183)
Trimester 3	-336 (-780, 108)	-233 (-848, 381)	-954 (-1587, -320)	-1442 (-2287, -597)
Entire Pregnancy	-1659 (-2240, -1078)	-905 (-1655, -154)	-2289 (-3007, -1571)	-2175 (-3110, -1240)
Exposure Window	NO_3, only	NO_3, all species	SO_4, only	SO_4, all species
ExPTB				
Trimester 1	134 (0, 269)	173 (27, 319)	77 (22, 132)	78 (18, 137)
Trimester 2	-10 (-150, 130)	-33 (-183, 116)	70 (10, 129)	100 (35, 166)
Entire Pregnancy	340 (111, 569)	*	-44 (-169, 80)	*
VPTB				
Trimester 1	136 (-41, 312)	218 (29, 406)	208 (135, 280)	233 (153, 312)
Trimester 2	40 (-131, 212)	112 (-70, 294)	171 (100, 243)	190 (111, 269)
Trimester 3	229 (-11, 468)	253 (-6, 511)	1 (-94, 97)	-5 (-110, 100)
Entire Pregnancy	583 (323, 843)	523 (262, 784)	23 (-110, 155)	60 (-74, 194)
MPTB				
Trimester 1	-55 (-318, 209)	156 (-130, 441)	364 (253, 475)	429 (306, 551)
Trimester 2	-329 (-590, -68)	-257 (-536, 22)	275 (164, 387)	337 (214, 461)
Trimester 3	206 (-91, 503)	245 (-73, 563)	104 (-11, 220)	139 (11, 267)
Entire Pregnancy	392 (27, 758)	275 (-99, 649)	152 (-24, 328)	244 (62, 426)
LPTB				
Trimester 1	175 (-335, 686)	793 (242, 1345)	551 (337, 765)	897 (661, 1133)
Trimester 2	-519 (-1030, -8)	-30 (-576, 517)	802 (584, 1020)	1118 (878, 1359)
Trimester 3	222 (-325, 769)	443 (-142, 1028)	389 (177, 602)	574 (339, 808)
Entire Pregnancy	1174 (476, 1872)	992 (273, 1711)	432 (108, 756)	816 (479, 1153)

*Models did not converge.
